# Understanding the nature of health: New perspectives for medicine and public health. Improved wellbeing at lower costs: New Perspectives for Medicine and Public Health: Improved Wellbeing at lower Cost

**DOI:** 10.12688/f1000research.7849.1

**Published:** 2016-02-12

**Authors:** Johannes Bircher, Eckhart G. Hahn

**Affiliations:** 1 Department of Hepatology, University of Bern, Meikirch, Switzerland; 2 Department of Medicine 1, University Hospital Erlangen, Erlangen, Germany

**Keywords:** Definition of health, Meikirch model, innovative health policies, potentials for health, health as a complex adaptive system, responsibility for health, social aspects of health, culture of health, health economy

## Abstract

**Background: **Current dilemmas of health care systems call for a new look at the nature of health. This is offered by the Meikirch model. We explore its hypothetical benefit for the future of medicine and public health.

**Meikirch model:** It states: “Health is a dynamic state of wellbeing emergent from conducive interactions between individuals’ potentials, life’s demands, and social and environmental determinants.” “Throughout the life course health results when an individuals’ biologically given potential (BGP) and his or her personally acquired potential (PAP), interacting with social and environmental determinants, satisfactorily respond to the demands of life.”

**Methods: **We explored the Meikirch model’s possible applications for personal and public health care.

**Results: **The PAP of each individual is the most modifiable component of the model. It responds to constructive social interactions and to personal growth. If an individual’s PAP is nurtured to develop further, it likely will contribute much more to health than without fostering. It may also compensate for losses of the BGP. An ensuing new culture of health may markedly improve health in the society. The rising costs of health care presumably are due in part to the tragedy of the commons and to moral hazard. Health as a complex adaptive system offers new possibilities for patient care, particularly for general practitioners.

**Discussion: **Analysis of health systems by the Meikirch model reveals that in many areas more can be done to improve people’s health and to reduce health care costs than is done today. The Meikirch model appears promising for individual and public health in low and high income countries. Emphasizing health instead of disease the Meikirch model reinforces article 12 of the International Covenant on Economic, Social and Cultural Rights of the United Nations – that abandons the WHO definition - and thereby may contribute to its reinterpretation.

## Introduction

Over the past century, biological and medical sciences have accumulated an enormous amount of knowledge and expertise. Today, the understanding of biological processes ranging from genetic mechanisms to organ function of complex living systems is huge. Much of it has been made available to individual and public health in order to prevent and treat diseased humans. As a result the current state of personal health can be seen as a major accomplishment. Life expectancy in high income countries has approximately doubled in the past 130 years (e.g., in Switzerland from 40.5 to 82.5 years) ^1^. Nevertheless, non-communicable diseases remain a major target of the World Health Organization (WHO) ^2^. The years lived with disabilities increases progressively and multimorbidity augments the demands on health care systems ^3^.

One way to further improve the current situation may be to clarify what health truly is, i.e. to offer a valid concept of health that can be applied to the care of individuals and to public health. For this purpose a new definition of health, the Meikirch model, was developed and published in 2014 ^4, 5^. As a result there are now firm theoretical indications about what might be achieved once the model is implemented. This paper has the purpose of initially briefly explaining the Meikirch model. Thereafter, investigations of its possible consequences focus first on the special role of the personality of each individual for his or her own health and shows how it may be supported further. Subsequently, the social and environmental determinants of health are reviewed in the light of the Meikirch model. E.g. development of a culture of health may substantially improve the health of whole populations. The rise in health care costs may be reversed because the Meikirch model counterbalances the tragedy of the commons. Health explained as a complex adaptive system offers new opportunities, particularly for general practitioners and rehabilitation institutions. We feel that putting the Meikirch model into practice could result in significant further world-wide improvements in health with decreased rather than increased costs.

## Meikirch model ( Figure 1)

The wording of the definition of health given by the Meikirch model is as follows ^4, 5^: “Health is a dynamic state of well-being emergent from conducive interactions between an individual’s potentials, life’s demands, and social and environmental determinants.” “Health results throughout the life course when an individual’s potentials – and social and environmental determinants – suffice to respond satisfactorily to the demands of life. Life’s demands can be physiological, psychosocial, or environmental, and vary across individuals and contexts, but in every case unsatisfactory responses lead to disease.”

**Figure 1.**
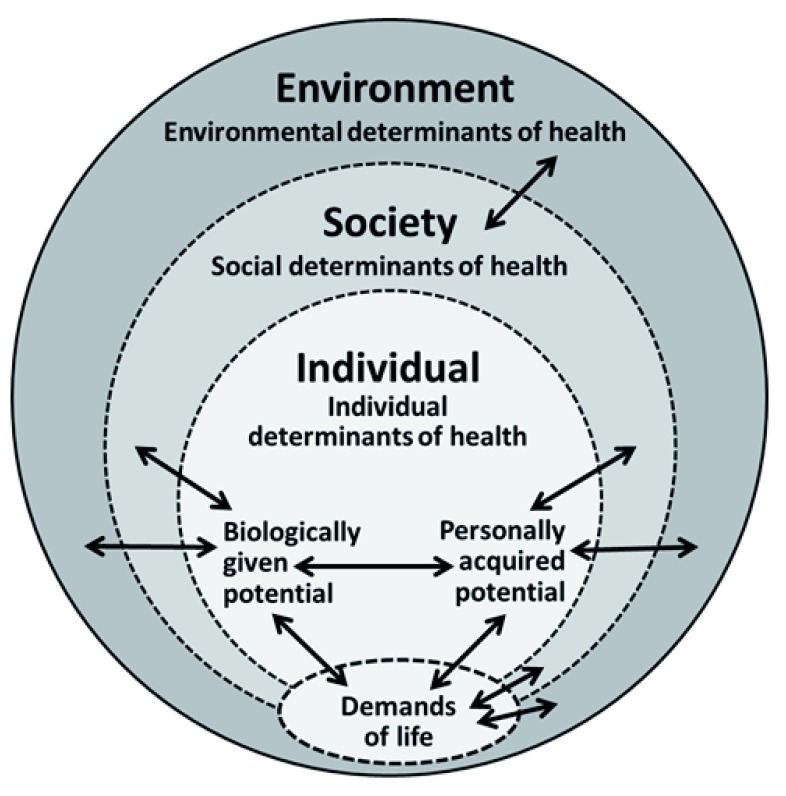
Graphic representation of the Meikirch model consisting of five components that are related to each other by at least ten complex interfaces (double arrows).

With this in mind, the Meikirch model consists of five components and ten complex interactions. These are shown in Figure 1 and Table 1, where the five components are depicted and the interactions are exhibited as double-edged arrows. The complete description of the model with its scientific background is given in the original publication. Here only a short explanation of the five components and their interactions is presented.

**Table 1.** Examples of interactions between the different components of the Meikirch model. (Abbreviations: BGP = biologically given potential and PAP = personally acquired potential).

Interaction between	Health Process	Function for Health	Purpose of Interaction
demands of life and both individual potentials	Life´s demands must be mastered to maintain life and health	Together the two potentials must successfully fulfil the demands of life.	To fulfil an essential requirement of life.
BGP and PAP	Development and maintenance of integrity of individual (Image: like horse and rider)	The PAP must assume responsibility for the BGP. It also can compensate in part for defects of the latter.	Development and maintenance of personal identity and resources is a condition needed for life.
BGP and social determinants of health	Development and maintenance of BGP	A life-affirming use of the BGP supports its development and its maintenance. Accident prevention and insurance may help.	Development and maintenance of the physical body throughout life.
PAP and social determinants of health	Development and maintenance of PAP	Life-affirming interactions create favourable conditions for the development and maintenance of the PAP.	Development and maintenance of the personality including self-responsibility and health leadership.
Social and environmental determinants	Interaction to preserve conditions for health	Environment may support or damage health by exhibiting life-affirming or life-denying conditions.	Alignment of environment with the determinants of health.
Social determinants of health and demands of life	Regulation of the demands of life	Social determinants of health may increase or decrease the demands of life.	Demands of life adapted to each person support health.
Environmental determinants of health and demands of life	Regulation of the demands of life	Environmental determinants of health may increase or decrease the demands of life.	Demands of life adapted to each person supports health.
Environmental determinants and the two potentials	Setting humans within environmental contexts	Human health is dependent on the environment and needs to take good care of it.	Humans need to adjust to the environment and to protect it.

### Life’s demands

Humans, like all other biological creatures, are exposed to the demands of life ( Figure 1). Their fulfilment is a condition for life ^6^. In people, these demands are physiological, psychosocial and environmental.

***1. Physiological demands.*** Humans have to meet physiological needs that vary with time and circumstances. They present themselves in many ways related to input, output, homeostasis, work, and procreation. “Intake of oxygen, nutrients and water, excretion, fertilization, pregnancy and childbirth are key examples. Some specific characteristics differentiate humans from other higher animals, e.g., the choice of whether or not to procreate” ^4^.

***2. Psychosocial demands.*** “Psychosocial demands relate to individuals’ personal development and social integration, including participation in the social, economic and political life. All these are interlinked. Each individual is exposed to various social determinants of health throughout the life course, with varying roles and expectations, as related to jobs, relationships, obligations to family and society, personal aspirations and political and economic contexts” ^4^. The way in which life’s psychosocial demands present themselves and can be fulfilled depends on the specifics of the respective individual and society. The final psychosocial demand is to achieve peace with the fact that every human must die.

***3. Environmental demands.*** Environmental factors may decrease or increase the demands of life in many ways, thereby affecting personal health. Examples are availability of clean drinking water, conditions for food production, air pollution, radioactivity and safe workplaces. In addition, protection from physical, chemical, and microbiological threats and safe disposal of waste matter (recycling) is essential. Some of these are apparent immediately, while others could be latent for many years (e.g., exposure to carcinogens from tobacco smoke, pollutants or radioactivity).

### Potentials

Each person needs resources to meet the demands of life. They have to satisfy the demands both in the present and in the long term. In order to serve the purposes of the Meikirch model the term potential was introduced to express both present and future resources. The potentials of an individual person have a double nature, parts are given and parts are acquired. Individuals always draw simultaneously on both potentials to meet life’s demands as outlined below.

***Biologically given potential (BGP).*** The BGP represents the biological basis of life. At the moment of birth it has a finite value resulting from genetic equipment, epigenetic regulation and quality of the pregnancy. This is the gift of life everybody receives. This potential diminishes naturally throughout life, reaching zero at the time of death. Every substantial social disadvantage, somatic disease, injury, or defect diminishes the BGP either transiently or permanently.

***Personally acquired potential (PAP)*.** The PAP is the sum of all physiological, mental, spiritual and social resources a person acquires during life. Presumably it starts to develop *in utero* while the baby hears the heart beat and the voice of the mother and feels her bodily movements. After birth, as the brain and other organs mature, this potential increases rapidly. Interactions in families, schools, and communities play a pivotal role for children and adolescents in supporting their acquisition of knowledge and skills and personal development. Even in adulthood the development of the potential continues, provided an individual cares for it. The social determinants of health have a very important enabling or inhibiting function. Integration and participation in the society is of outstanding importance for each individual. The society also provides many other helpful possibilities, such as work, food shops, health information, social security, and a health care system. Finally, the environmental determinants of health exhibit decisive enabling and threatening functions for the PAP of each individual.

### Social determinants of health

Social determinants may be supportive or challenging for people’s health by modifying the demands of life and by supporting or hindering the development of their potentials. This starts in families and continues in schools and during professional formation ^7, 8^. These influences result from interacting individuals or as a result of the prevailing culture in the society. Throughout life social concerns, mutual trust, and collective efficacy support a good health outcome. Wilkinson and Pickett identified better health of people in countries with less inequality of incomes ^9^. In many parts of the world the social determinants of health are not optimal. Poverty, difficult living and work conditions may limit the health people can achieve. “Longevity is not solely related to people’s income, but is also strongly affected by their autonomy and social participation, which are major determinants of health” ^8^.

### Environmental determinants of health

In 1987 the UN World Commission on Environment and Development’s report, Our Common Future, also known as the Brundtland report, noted: The "environment" is where we all live; and "development" is what we all do in attempting to improve our lot within that abode ^10^. Factors in living and work environments may not be cared for sufficiently, e.g. global warming ^11^. Also coal mining is an important environmental cause of diseases as are water-borne contaminants. Use of antibiotics in agriculture has contributed to human infections with resistant germs. “Adopting cleaner, more sustainable energy technologies and water sources could help promote both health and development. At the macro level, dwindling natural resources, population growth, and the effects of climate change are likely to affect global health” ^4^.

### Health as a complex adaptive system

A complex adaptive system is an entity with a boundary between it and its environment, that can take up material and energy from the environment (input), release end products (output of entropy) and do work ^12– 14^. Within the system there are many different parts called agents. They continuously interact with each other in a nonlinear manner, resulting in an outcome, called emergence, that is more than the sum of the properties of these agents. In the Meikirch model the five components, including their subcomponents, are regarded as agents ( Figure 1). They spontaneously and autonomously arrange themselves in such a way that the evolving products are an emergent expression of the functioning of the system as a whole. These arrangements are operational, but are not necessarily the best solution for the system. Nevertheless, in complex adaptive systems, including humans, emergence is definitely more than the sum of the properties and functions of the parts.

In a complex adaptive system there is a flow of energy. Via their inputs all living organisms take up energy from their surroundings. Humans have within themselves an additional source of energy, e.g. vitality, drive and sense of purpose ^15^. One part of the total energy is spent to maintain the person physically and emotionally and another part to do work. The material output (excretions) contains the end products and represents entropy. The flow of energy in human beings also leads to a desire for being loved, pursuing values, and living for a purpose. Investigating the double nature of this energy flow of an individual helps to better understand her or his state of health.

## Methods

For the purpose of this investigation the Meikirch model was used as a basis to investigate selected possibilities to positively influence personal and public health as described in the Results section. In each instance two questions were asked: Does the examined situation agree with or contradict the Meikirch model? If it agrees, does the Meikirch model offer new perspectives for individual and/or public health? The deduction/induction cycles about the findings were applied repeatedly until the results appeared to be consistent.

## Results

The Meikirch model is designed to support a new, unified approach to health and disease under various conditions in diverse health systems. Some results of such an approach are delineated in the following section:

### Modifying the demands of life

Environmental determinants of health have impacts on the two human potentials and on the demands of life ( Figure 1). Geography may dictate, for example, how and which type of food can be produced, and how housing and clothing has to be. In addition, the demands of life vary with the social determinants of health. They differ in low income and high-income countries. Demands also vary from person to person and in different cultural backgrounds. In some places women are primarily concerned with raising children, whereas men care for food production. In others the tasks are different. But role models change continuously. It often appears that not much can be done to modify the demands of life. For young individuals, therefore, it is important to choose wisely among them and, if possible, get away from life conditions that are detrimental to health. In the future, for a given cultural background, this might be reflected in appropriate educational programmes.

### Care of the biologically given potential (BGP)

After a healthy pregnancy the BGP at birth is a gift of nature and its vitality may vary from person to person. Thereafter it must be cared for. Social factors may foster or curb physical exercise, quality and quantity of food, alcohol intake, smoking, and consumption of narcotic drugs. These features are well known in pedagogy.

In earlier times epidemics have wiped out large portions of populations. Acute and chronic disease burdens threatened the BGP. Today, improved social determinants of health, e.g., public health and medical programmes, have achieved healthy lives and long life spans for a majority of the people in high income countries whereas low-income countries are still concerned with further developing their public health and medical services. Everywhere economic factors limit in one way or another what can be done for the BGP.

### Promotion of the personally acquired potential (PAP)

The PAP is presumably the most fragile and modifiable component of the Meikirch model. It is subject to strong influences through interactions with other components of the model. The PAP is a critical factor for the achievement of satisfactory or unsatisfactory responses to the demands of life. Thereby it strongly influences the realization of health or disease. Consequently, it is of greatest interest to review the factors that may promote or impede the development of this potential.

***1. Important factors that modify the PAP.*** In each individual this potential is small at birth, yet grows rapidly thereafter. From the first day children mature with the love and care of their parents ^19^. Later, teachers play important roles. Eventually individuals have to assume responsibility for their PAP themselves. Yet, they remain affected by their social settings. Although every person continuously has to contribute him- or herself to this potential, it may be supported further by trusting, loving, respectful, and life affirming human relationships. A well-founded sense of purpose in life strengthens it also ^16^. Alternatively it may be damaged by stress, adverse social circumstances like educational deprivation, sexual exploitation, and abuse of alcohol and narcotic drugs. Theoretically, at any moment, each individual has choices about how to handle his or her life situation. In order to choose from the existing possibilities as wisely as possible, each person develops more or less personal leadership ^17^. Thus, the PAP of any individual is the result of complex interactions between the respective personality and his or her social settings.

***2. A high PAP is important for health.*** The PAP is critical for the choices an individual makes about how to approach the demands of life. Affinity for certain types of work, ambitions, vitality, attitudes toward rewards, etc. will influence the choices of and dedication for work. These aspects apply equally to work at home, including attention to children as well as to work outside the home, e.g., professional contributions to the society. Ideally the specifics of each work situation are matched with knowledge and skills of each worker.

Attitude, culture, willingness to learn theoretically and from experience, and the possibility to grow with new challenges will have major influences on health. This is exemplified by several approaches: In his concept of salutogenesis, Antonovsky postulates that a high sense of coherence is essential for successful coping ^15^. Comprehensibility, manageability and meaningfulness are central features of this sense of coherence. Presumably they can be learned and life managed correspondingly. Also van Spijk postulates that leading a life that makes sense results in “human great health” ^18^. Martin Seligman found that positive psychology is closely related to health ^19^. Flourishing persons who experience more than three positive feelings for every negative feeling are healthier and live longer than languishing persons. In the Meikirch model this may be explained by the contribution of positive emotions to the PAP. Overarching positive feelings or positive self-perception strongly influence the approach to life as shown by the following two studies. Nuns with more positive emotions at the age of about 22 years survived six years longer than nuns with more negative emotions at the same age ^20^. “Individuals with more positive self-perceptions of aging lived 7.5 years longer than those with less positive self-perceptions” ^4, 21^. Other examples are given in Table 2. Methods to improve the PAP including its implication for health need to be investigated urgently and in depth.

**Table 2.** Twelve evidence based examples illustrating the close relationship between personally acquired potential (PAP) and aspects of health.

1. Brief daily yogic meditation of family dementia care givers reduced transcription of pro-inflammatory cytokines and increased IRF1-related transcription of innate antiviral response genes ^63^.
2. Hedonic and eudemonic well-being engaged the immune system with different gene regulatory programs suggesting that the human genome is sensitive to qualitative variations in well-being ^64^.
3. In the treatment of subacute and chronic low-back pain cognitive behavioural therapy was more effective and less costly than physiotherapy. (Pragmatic randomized controlled trial) ^65^
4. Multidisciplinary biopsychosocial rehabilitation interventions were more effective than usual care and physical treatments in decreasing pain and disability in people with chronic low back pain ^66^.
5. Cognitive behavioural therapy reduced the risk of recurrent cardiovascular disease or myocardial infarction ^67^.
6. Diabetics with inadequate functional health literacy presented with higher odds of poor glycaemic control ^68^.
7. In ten randomized controlled trials patients with impaired glucose regulation were followed. Progression to diabetes was reduced by lifestyle interventions by over half in some trials ^69^.
8. Among community-dwelling older adults, inadequate health literacy was independently associated with poorer physical and mental health ^70^.
9. Psychological distress is associated with increased risk of mortality from several major causes in a dose response pattern. Risk of mortality was raised even at lower levels of distress ^71^.
10. Empirical data from 2655 elderly people showed that extraversion, openness, agreeableness and conscientiousness traits were associated with better elf-perceived health ^72^.
11. At the end of life the PAP plays a major role. “Dignity therapy” resulted in decreased suffering and increased will to live. In addition life appeared more meaningful and with more sense of purpose ^73^.
12. Early palliative care provides both in and out of the hospital a better quality of life. Cost savings through reduced resource use are an epiphenomenon of this better care ^74^.

***3. Personal health leadership.*** In principle every person has considerable influence on his PAP. Consequently, in as much as humans are in control of themselves, it is necessary to consider that they also have to assume personal responsibility for their health. Correspondingly, they may or may not “lead a healthy life”. This popular expression implies that the importance of leadership for personal health has been known publicly for a long time. If so, everyone might be considered as an entrepreneur of his or her own health. This is an encouraging, positive empowerment for health that may motivate people to invest in it. This idea as expressed in the Meikirch model is now being practised successfully in indigenous villages in Odisha, India ^22^. The essence of the model was easily understood even by analphabetic persons and it changed their behaviour into a much more health supporting pattern. Until now the idea of personal responsibility for health has not received sufficient attention. This presumably is due to the fact that in the past the origin of many diseases has been unexplained and therefore diseased humans were not to be made responsible unjustly. In 2003, however, WHO has launched its campaign against non-communicable diseases ^23^. These conditions are thought to be largely preventable by health protecting behaviour. This justifies the concept that all humans are called to assume personal leadership for their health.

***4. Education for a culture of health.*** Leadership for one's PAP might further be reinforced by introduction of a culture of health ^24^. For this purpose all professionals of public health and individual medical care might be involved. They should teach about the Meikirch model and the relevant health conditions to students and lay persons from kindergarten up to professional maturity and old age. The principles for transformative learning worked out for health professionals may be applied equally to the general population ^25^. For example teaching patients with type 2 diabetes improved their health ^26^. Schools, universities, and the media should play an important role. Voices and actions of public role models might also be encouraging. In addition to the Meikirch model, easily understandable information about prevention and treatment of prevalent diseases appears to be essential for the general public. A central organization e.g., a “National Institute of Health Information” might be made available as a library and reference centre of trustworthy health information for the whole population. Once a new culture of health is established, an impressively improved health of the people most likely will be the result.

***5. Vaccination and levels of control of infectious diseases.*** The grave lack of a culture of health is well illustrated by diseases such as poliomyelitis, and tuberculosis. In theory, poliomyelitis could be eradicated like smallpox by mass vaccination for all the people in the world or by surveillance containment strategies; in practice, concerted efforts by the WHO had it difficult to find acceptance in some countries, and major resurgences occurred in India and Nigeria, reintroducing polio virus type 1 into more than 20 previously polio-free countries ^27^. By adequate treatment of patients infected by *mycobacterium tuberculosis*, tuberculosis could be controlled. But, being essentially a disease of poverty, deliberate efforts and continued improvement of social determinants are necessary but were difficult to achieve, particularly in poorer countries ^27^. Although the WHO has made major efforts in this direction and has had much success, without mutual trust and support resulting from a culture of health embracing each and every person these goals probably cannot be attained.

***6. Relationship between the two potentials.*** Interestingly, the proposed type of health leadership and self-management also applies to cultivating the complex relationship between the two potentials (BGP and PAP). An analogous relationship exists in the bond between a horse and its rider. If the rider wants his horse to serve him well, he must take good care of it. The horse needs cleanliness, adequate food and water, an appropriate amount of physical movement, and rest at night in a protected area. In addition, horse and rider must trust each other and the rider must remain in control. Although this analogy with the relationship between a person and his body may be surprising, it illustrates that the two potentials must remain at one with each other. Further features render this relationship important: To a significant part the PAP can compensate for defects of the BGP. This ability is used in rehabilitation. In some conditions it may go quite far. E.g. paraplegic patients may again become independent and professionally active ^28^. When asked, they may even say that they are “healthy”. The compensation of defects in the BGP by the PAP is also important for elderly persons who experience increasing somatic defects. Yet, after having learned to adjust to them, they may experience a sense of well-being and lead a very satisfactory life.

### Social determinants of health

Social determinants of health are of fundamental importance ^29^. Among other aspects they are complicated by the fact that health care also has an economic component. This has been well known since antiquity. Today, health economy has a much higher and ever increasing impact because health care systems have become more and more costly.

***1. Epidemics.*** The relation between social determinants of health and the PAP becomes vital in case of epidemics, as exemplified by the Ebola crisis in West Africa in 2014 ^30^. It clearly illustrates the needs for careful investigation of the disease itself, interruption of its transmission, and care of the diseased persons. This means that people have first to understand the disease and what they must do for prevention and for treatment of diseased individuals. They must then become convinced of the purpose of what they do. Last but not least they have to receive the equipment and support they need to become effective. To achieve appropriate handling of diseased individuals, a corresponding organization is essential, particularly because in most cases cultural limitations may have to be overcome. This requires appropriate educational interventions and intense interactions between the responsible organization and the population. An educational programme based on the Meikirch model may be very useful, because it explains the importance of a PAP and gives all participants an identical view about how social determinants of health contribute. Successful cooperation requires agreed upon, common objectives ^31^. The Meikirch model might be most appropriate for this purpose.

***2. Tragedy of the commons.*** In high-income countries many current health care systems offer various services at no or minimal extra costs beyond the ordinary premiums for health insurance. For Germany, the OECD has documented 9.9 average “episodes of care” versus 6.6 for the OECD average of 33 countries, which is the highest in Western Europe. This is not limited, and for each “episode of care” multiple physician contacts are involved ^32^. Patients with compulsory insurance had a mean of 18 physician contacts per year in 2007 (8 physician contacts in the age group 11–15 years; 37 physician contacts in the age group 81–85 years) according to one large insurance company ^33^. Although it is difficult to interpret such statistics, they document overuse in a wealthy country. Hardin has compared such situations to the so-called “tragedy of the commons” ^34^. Whenever a utility is freely available to everybody and paid collectively, it follows logically that everybody tries to get as much out of it as possible. This is no problem as long as the utility is available in excess. The tragedy begins only once the utility becomes the limiting factor. In the past this occurred when farmers used the commons to feed their cows. Every farmer added one or two cows to his allowance. Overgrazing resulted in the tragedy of insufficient feed at the end of the season: the so called “tragic depletion”. Interestingly there is no technical solution to this problem. The tragedy only stopped once the commons were distributed among the farmers as property and each of them had to be satisfied with the amount of land he disposed of. Such a change is, according to theory, often prevented by the “tragic stalemate”: nobody wants to do the first step ^35^. Today such arguments may be applied to the use of health care services, as was observed for the USA ^36, 37^. In analogy, the continuous rise in health care costs will not be stopped by appeals and rationing will produce undesired effects. It will become limited only when each patient establishes ownership of his or her health and will assume more responsibility for his or her own health care, including financial consequences. Such a condition might further strengthen health leadership as explained above. Implementation of financial responsibility of individuals for their healthcare, however, will be difficult to introduce. Obviously it must be developed by trial and error without undue hardship for socially disadvantaged groups. Health economists, ethicists and politicians will have to work together. The Meikirch model may facilitate convergence of more or less affluent countries as recommended by the WHO Commission on Social Determinants of Health ^29^.

***3. Influence of health care payment models on physician work practice and professional satisfaction of all involved participants.*** Payment models are designed to produce financial incentives in order to achieve certain goals. Examples are capitation, episode-based and bundled payment, shared savings, pay for performance (PFP), and retainer-based practice ^38^. They are designed from different points of view e.g. to “improve patient care, to preserve or enhance physician professional satisfaction, to satisfy multiple external stakeholders, or to maintain economic viability as business” ^39^. Porter’s proposal e.g. intends to improve “value” in health care ^40^. The proposal is formulated from the point of view of the payer. Other actors in the health care systems may have different points of views ^41^. The most important concern is that payment systems may be manipulated for personal gains. Yet, once patients no longer are sure that their physicians act exclusively in their best interest, the patient-physician relationship is strained ^42^. Today there are many examples of manipulation. Bernard Lown *et al.* ^43, 44^ have shown this phenomenon in cardiology, but other fields of health care are equally vulnerable. The Meikirch model offers a new point of view. In each individual patient the primary aim of a payment system should be to optimally support improvement of health as defined by the model. If all interested parties share this objective, payment systems may be worked out that fully protect the patients interests, serve all partakers, and thereby also are cost-effective ^45^. If these conditions are fulfilled, the payment systems also will lead to professional satisfaction of physicians and other interested parties.

***4. Moral hazard.*** Moral hazard may occur when insurance payments change the behaviours of patients or physicians. This is possible for cases with diseases or treatments that are difficult to assess objectively. Such situations may therefore be exploited by patients in order to get payments ^46, 47^. Independent medical evaluations for social security eligibility may then become necessary. The Meikirch model was found to be useful also for such evaluations ^48^. As outlined above, moral hazard undermines mutual trust between diseased persons and physicians. A shared vision of health as set out by the Meikirch model may make it easier for physicians to improve performance and accountability and for patients to truly agree on indications, e.g., for expensive examinations and for surgery ^40, 41^.

***5. Stressful working conditions.*** Social determinants contribute to both the demands of life and the two potentials ^49^. For instance, during the industrial revolution the majority of employees had to work very long hours and often in unhealthy settings. Unfortunately, in many low or middle-income countries, such practices have not as yet been stopped. Working conditions evolving from a continuous mutually supporting dialogue between the workers and management may render engagement in a working place more interesting, more flexible, more rewarding and particularly also more productive ^9^. “Economists have recognized that good communication between workers and management, leads to better working conditions and may result in important productivity gains while simultaneously reducing costs ^50^. If such communication includes the Meikirch model, health of the employees might be improved and health care costs reduced.”

### Environmental determinants of health ^11, 51^

The relationship between social and environmental determinants of health offers another excellent example of the tragedy of the commons. The environment is freely available. Therefore in many places it is used without restrictions and poisoned correspondingly. When the population was small, waste created no problems, and nobody had to pay for it. Population growth, technological developments, and increase in wealth, however, have now led to major problems. In Switzerland, for example, most lakes were polluted to an extent that life in the water became almost extinct. When waste water was cleaned before it was allowed to flow into the lakes, water quality was much improved. Today, a worldwide overproduction of CO _2_ and other waste products increase greenhouse gases to an extent that the temperatures of the planet will rise. Atomic power plants create an analogous situation. Everybody needs electrical power, yet nobody knows what to do with the atomic waste. In addition, the Fukushima catastrophe has again revealed how dangerous accidents in atomic power plants may be. A further example is exploitation of some fish populations that led to their extinction. Pollution of air, water and soil has serious consequences in many places. Therefore, for the purpose of health, many new agreements and their strict enforcement will be required in order to correct the current untenable situation.

### Health and disease as states of a complex adaptive system

Investigation of the state of health of an individual by a systems theoretical approach is as yet in its infancy, but scientific methods based on Newtonian physics are inadequate ( Table 3 and Table 4) ^12, 13^. A new approach should involve individual health as a whole ( Figure 1), should consider its history, its energy flow and its sense of purpose.

**Table 3.** Comparison of natural science with complexity science concerning health.

Natural science	Complexity science
Thinking is based on reductionism taking Newton’s physics as model.	Thinking is based on systems theory and on holism.
Attention focuses on mean values.	Attention focuses on variations.
Control is Top-down.	Control is peripheral and Bottom-up.
The organism is put together from parts.	The organism results from morphogenesis.
Determinism means that further evolution of the organism may be predicted from initial conditions.	Indeterminism means that further evolution and emergence occur autonomously and cannot be derived from initial conditions.
The different parts function independently of each other.	The components of the system are called “agents”. They interact nonlinearly with each other in a complex manner. They function together as a whole.
Predictable (e.g., linear or Michaelis-Menten) relationships mean that changes occur continuously.	Nonlinear relationships also include critical thresholds.
Changes are centrally controlled.	Changes are influenced locally.
Observed effects are the direct results of the influence of one object on another reflecting the properties of the objects.	Observed effects result from repetitive feedback and lead to a change in the organization and emergence of the system. Emergence cannot be explained by the properties of the agents.
Evolutions and results of treatment are predictable	Sensitivity analysis may improve the understanding of possible evolutions. Emergence may be either as hoped for or completely new.

**Table 4.** Newton based science and complexity science applied to the practice of medicine.

Feature considered	Newton based science	Complexity science	Implementation of complexity science in medical practice
**Type of thinking**	Analytical reductionism	Holistic, narrative, empirical, evolutionary, phenomenological	Patient is respected as an independent self- governing individual.
**Metaphor**	The current organism is an assembly of necessary parts.	The current organism has evolved from prior states and will evolve further, yet its future is not determined.	Health is viewed as a dynamic state that responds to influences at all levels.
**Nature of subunits**	All components function as discrete entities and perform what they are made for.	Components are agents. They have specific functions and continuously interact with each other in a complex manner	The Meikirch model has five components that cannot be removed like the appendix, repaired like a heart valve, or replaced like a hip by arthroprosthesis.
**Relationship among** **entities**	Linear or otherwise known relationships	Linear and nonlinear relationships including critical mass thresholds	In the Meikirch model relationships are complex: Multiple aspects interact with each other.
**Past history**	Relatively unimportant	Essential for an understanding of the presenting condition	Interest for the history has always been a feature of medical practice.
**Type of control**	Top down: self-responsibility	Bottom up: self-organization	The patient wants to be respected and understood as an independent entity, but not to be controlled.
**Assessment**	Diseased parts deviate from statistical mean and normal range of functions.	History may be used as sensitivity analysis: How were past crises managed and with which results?	History focuses also on the narrative of past crises and tries to understand destructive and constructive features of each.
**Performance**	Predictable, depending of the performance of the weakest part	Emergence is a new quality which cannot be derived from the properties of the parts.	Antonovsky’s sense of coherence consists of understandability, manageability and meaningfulness. The physician will consider individual components.
**Importance of ends**	Goals are not needed.	Systems function best, if they pursue goals or objectives.	Constructive experiences from the past and Antonovsky’s meaningfulness are important.
**Treatment**	Successful correction of abnormalities consistently results in cure.	A new, possibly “healthier” state may emerge from internal rearrangements related to changes of objectives and/or the environment.	A mutually trusting patient-physician interaction is central. The physician must believe in the patient’s ability to evolve to a new state and support it. Follow-up interactions are important.
**Prognosis**	Predictable	Unpredictable	Unpredictable outcomes will be expected and taken into account by personal care.

As a first approximation the system’s approach comes close to the best of the old fashioned medical history. The patient is to be carefully asked at least the following questions: How was life before onset of the disease? What were the weaknesses and the strengths? How were the conditions around the time of the first symptoms? What was new? What were possible triggering or helping factors at that time and also during further episodes of the disease? How did the patient’s sense of coherence (understandability, manageability and purpose) evolve during the disease? ^15^ What was the purpose in life, and what is it now? How does the patient himself interpret her or his symptoms or condition? How did the patient respond to the different stages of the disease? What could be the most appropriate evolution for the patient and how does she or he see it? These questions may then be modified to try to assess the energy flow, including energies driving preferences of behaviour, values and spirituality. Specific questions about the purpose in life may inform about some of these aspects ^14, 52^.

Systems methods may particularly be applied by general practitioners, because very often they have known the patient for years, are also aware of his or her social network and have gone with him or her through previous crises. A long-standing and trusting patient-doctor relationship is a most powerful diagnostic and therapeutic tool ^53^. In many cases modern diagnostic procedures, expensive drugs or seemingly appropriate surgical interventions can be avoided, because they involve risks and encompass unpredictable reactions of the patient’s system. Such restraint may be wise and prevent complications. Reorientation of the patient’s intentions and sense of purpose may induce healing processes. Active participation of the patient is important. Similar considerations apply in rehabilitation institutions, where patients may be observed closely ( Table 4).

This brief allusion reveals that at the present time system’s thinking in medicine is at its very beginning. For appropriate treatment of the PAP much further diagnostic and therapeutic research is needed. Systems theory has a good chance to evolve to a very powerful tool in the coming years.

## Discussion

At the present time the Meikirch model ^5^ is just a hypothesis with new possibilities and new limitations. For these reasons all postulates in this paper await an in depth scientific confirmation. Nevertheless, conclusions derived from the model are supported by observations, logic, and literature giving them already now some validity. Most notably, the Meikirch model fulfils the postulates expressed by the meeting of experts reported by Huber *et al.*: “ability to adapt and to self-manage” ^54^.

The concept of health as a complex adaptive system may raise the question as to how medicine has functioned so far without reference to the systemic nature of health. A partial answer may be as follows: Even treatments that are established as being effective do not give the same results in all patients. Different individuals respond in diverse ways to exactly the same procedures. Controlled clinical trials are thus needed to decide about the overall effectiveness of a treatment, but they also show how much individuals may deviate from the mean. To translate study results into decisions for individual patients it has always been an established practice of physicians to carefully consider medical histories and to follow up each patient in order to recognize possible developments in an undesirable direction. Systems theory gives these empirical practices a theoretical basis.

When looking at the Meikirch model it appears that the PAP offers great opportunities to do much more for health than has been done so far. Since the publications of Virchow in 1858 health and disease were considered to be properties of the physical body ^55^. There were some good reasons for this assumption: Ordinary infectious diseases, many cancers and the sequelae of accidents have clear physical, chemical, or microbiological causes and manifestations. Whenever these causes could be eliminated, corresponding improvements in health were the result. On the other hand physicians are aware that many patients suffer from important symptoms that cannot be explained adequately by objectively recognizable signs of disease. Others have distress that is distinctly more severe than in most patients who suffer with the same physical condition. There are also patients who do not complain even though they are severely diseased. These observations support the concept that the state of health of each patient should truly be regarded as a complex adaptive system. This feature allows a new look at symptoms and offers new possibilities for treatment. For example, solutions found in past crises may teach the patient and the physician new therapeutic options for current problems. In some cases diseases may serve as a learning opportunity on how to conduct one’s life. We also think that psychological support for the creation of a new future for a patient may have a high therapeutic value. A positive belief of the physician in the patient's abilities may be very valuable to readjust himself with his whole system toward a better state of health.

An interesting new aspect of the Meikirch model is that it may serve as a joint vision for all people who are personally or professionally concerned with health, be it patients, physicians, nurses, administrators, health workers in any profession, journalists, business people or politicians. A common goal is particularly important for departmental, interdepartmental and intersectoral cooperation. In the future, a shared vision for all people based on the Meikirch model may be a great asset that strengthens public health and medicine ^31^. The model may also serve well to interpret article 25 of the Universal Declaration of Human Rights (1948) ^56^ and article 12 of the International Covenant on Economic, Social and Cultural Rights (1976) ^57^. Use of the five components and ten complex interactions may help to better understand the social structure of some health problems. A good example is the increasing prevalence of patients with burnout. This condition is initiated by current types of management procedures designed to stimulate employees to the extent that they give their maximum or even more. Consequently, their demands of life are pushed up as much as possible and a rising number break down. Thereafter health and social systems have to take care of them. This implies that one part of the society, i.e., business, creates a problem and another part of the same society, i.e., social security, has to pay for it. With the help of the Meikirch model such unethical situations can be recognized easily and may lead to corrections that obviously must occur at a political level.

Our observation is that today’s health care systems have in part been distracted from their main mission by rising health care costs. This corresponds surprisingly well to the tragedy of the commons ^34^. It applies equally to patients and physicians and leads to excessive amounts of investigations and operations ^44^. Furthermore, moral hazard may aggravate the difficulties ^46, 47^. All parties involved should be reminded that initially, i.e., in the past, they made a contract for health and not for a maximum of services or of income. How to balance health care with limited financial resources is an interdisciplinary dilemma that cannot be solved by individual health workers, administrators, or patients. In each context policies that successfully combine scientific, economic, and normative postulates must be worked out among all stake holders. In the past, the lack of a common concept of health limited the success of such interdisciplinary and intersectoral groups. We trust that the Meikirch model will improve procedures to solve these problems.

Some confusion has occurred in the past about the importance of the relative contributions to people’s health by public health activities or by individual medical care. Unfortunately, mutual awareness and respect between the two approaches to health have remained limited. Interestingly, such situations may be understood better by “Russell’s paradox” ^58^. Bertrand Russell found that the combination of observations at two different levels may lead to incompatibilities. A good example for an intuitive understanding is the following statement: “The world is bad! Everybody cares exclusively for himself! Only I care for myself!” In the health care system Russell’s paradox becomes apparent when looking at parameters such as health and disease: When investigated by the Meikirch model an individual can be healthy or diseased. In contrast, most groups of people or populations can neither be healthy nor diseased. Only a portion of the investigated individuals is either healthy or diseased. Therefore public health works with indicators of health or disease such as longevity, live birth rate, mortality rate, etc. and expresses them in statistical numbers. Interestingly, a public health intervention such as vaccination in a group of people is likely to reduce the prevalence of the disease. Yet some individuals may contract the disease and will not experience the positive effect of their vaccination. Consequently vaccination is effective when analysed in groups, but not necessarily in individuals. The patient-physician interaction is important in individual medicine, but not in public health. We conclude that mixing judgments of individual medical care with judgments of public health - including comparisons of their relative contributions to the health of a population - is not purposeful and must be avoided. Indeed, they simply complement each other. Some reluctance to support the Meikirch model in the health literature may presumably be explained by Russell’s paradox ^59– 61^.

The Meikirch model implies that for each individual demands of life and two potentials are critical for health. The high interest in natural sciences has led to an impressive machinery for the improvement of the BGP. In contrast, the PAP with its non-technical aspects has by far not received the same attention. We conclude that current medicine and public health will be able to offer much more health support, once the importance of the PAP is generally recognized, further developed, and fully integrated into all health care activities. The Meikirch model offers completely new opportunities; this applies to diverse settings at local and global levels and in low and high income countries. This new view of health and wellbeing may also be interesting for the interpretation of the Universal Declaration of Human Rights ^56^, the International Covenant on Economic, Social and Cultural Rights ^57^ and the General Comment No. 14 related to art. 12 of the International Covenant ^62^. In principle the Meikirch model is applicable in any situation where a concern for health is relevant.
