# Supplementary material for: Understanding the nature of health: New perspectives for medicine and public health. Improved wellbeing at lower costs: New Perspectives for Medicine and Public Health: Improved Wellbeing at lower Cost
Source: F1000Res. 2016 Feb 12;5:167. [Version 1] doi: 10.12688/f1000research.7849.1 (PMC4837984; doi:10.12688/f1000research.7849.1)
Supplement: Peer review history

# Peer review: Referee response for version 1

**DOI:** 10.5256/f1000research.8450.r12424

**Published:** 2016-04-18

**Authors:** Joachim P Sturmberg

**Affiliations:** 1 School of Medicine and Public Health, University of Newcastle, Wamberal, NSW, Australia

*Understanding the nature of health: New perspectives for medicine and public health. Improved wellbeing at lower costs* is an attempt to highlight the interconnected nature of health between the individual and his “lived environment”. It is an important step towards reframing ^1^ the necessary societal discourse about the nature of health and its implications for personal and professional care and the organisation of healthcare in one’s society. The authors are commended for making these complex tasks understandable and manageable for patients, practitioners and policy makers. In particular they make compelling arguments why both users and providers of health care need to come together and “renegotiate” the purpose of healthcare and define mutual expectations from the health system.  

Following are a number of thoughts stimulated by reading the article for consideration in future revisions of this paper.

*(1) Understanding the nature of health*. There has been important other work done in this domain, e.g. Macklem ^2^, Jylhä ^3^, Jadad ^4^, Lewis ^5^, Sturmberg ^6^^,^^7^ and Sturmberg *et al* ^8^. Importantly the “experiential nature of health”, pointing to the importance of adding a cognitive domain to the definition of health, deserves more explicit mention. A review of the “health definition” literature highlights that the experiential aspect is persistent and central across time, philosophical traditions and cultures ^9^^-^^21^. The authors only refer to its importance in indirect ways:

 

- *The final psychosocial demand* [which in reality is a cognitive ability] *is to achieve peace with the fact that every human must die.*
- *A well-founded sense of purpose in life strengthens it* [personally acquired potential] *also.*
- *A high PAP *[personal adaptive potential] *is important for health. *
- *In some cases diseases may serve as a learning opportunity on how to conduct one’s life.*
- [health as an] *ability to adapt and to self-manage.*

 

The importance of the cognitive domain for health is also reflected in the development of subcultures in health, usually at the extreme of the spectrum like drug use as self-therapy for mental health experiences or the obsessive consciousness on health in every action of daily living.

Understanding health as “experiential and in one’s one context” is further reflected in health inequality. It is “relative” inequality that matters most, as stated in the paper:

 

- *The social determinants of health have a very important enabling or inhibiting function.*
-  
*Wilkinson and Pickett identified better health of people in countries with less inequality of incomes.*
-  
*Longevity is not solely related to people’s income, but is also strongly affected by their autonomy and social participation, which are major determinants of health.*

  

(2) *Economics*. While there is no doubt that economics play an important role in financing health, the statement

 

- *Today, health economy has a much higher and ever increasing impact because health care systems have become more and more costly.*

 

reflects a one-sided a view. It echoes the economic rationalist [linear] paradigm. In the context of this paper, it needs to be debunked. Principally a ledger has two sides – income and expenditure; more importantly though, what is an expense in one ledger also is an income in another ledger. Currently politicians, bureaucrats and financiers put forward rather one-eyed arguments to justify “cut and burn” approaches in the reform efforts of our healthcare systems. The “unintended” but highly foreseeable consequences are degradations of social and environmental conditions so necessary to the “production of good health” ^22^.

(3) *Greater emphasis? Epigenetics*. In the context of “dynamics of health” it appears essential to include the growing evidence of the important aspects of epigenetics and in particular those of social epigenetics ^23^. These insights are important in understanding variation in disease expression in various communities. Equally, they explain disease distributions in communities but more importantly that health improvement strategies may take a few generations to show reversals in disease distributions.

*Genetic and physiological network interactions*. There is a need to at least hint to the “low level mechanisms” that explain the observations between the environmental, social, emotional and cognitive perturbations on health ^24^^,^^25^. These insights satisfy “the scientific need of argument” for various players to engage in “system’s based” decision-making and strengthen the concluding remarks:

 

- *In each context policies that successfully combine scientific, economic, and normative postulates must be worked out among all stake holders.*

 

*The limitation of controlled trials*. The basic assumption underpinning controlled trials are inconsistent with “complex adaptive systems” frameworks. Complexity entails nonlinearity and variability, two features of nature that cannot be controlled for ^26^ making inferences from controlled trials tenuous. Tools like neural network or cluster analysis provide the insights that are really needed in personal care and population health decision-making.

(4) *Changing the worldview*. I think it is fair to state that “modern medicine” has lost its soul – medicine was always primarily about care rather than cure – just consider Archie Cochrane: *Cure is rare but the need for care is widespread* ^27^. The power of care, linking back to above mentioned “physiological networks”, is greatly underestimated and its importance is largely missing from the medical curriculum. The authors’ comments in this regard:

 

- *We also think that psychological support for the creation of a new future for a patient may have a high therapeutic value. A positive belief of the physician in the patient’s abilities may be very valuable to readjust himself with his whole system toward a better state of health.*

 

cannot be overemphasised. Care leads to self-care which ultimately leads to better “experiential health”, independent of the presence or absence of identifiable pathologies. It is right to emphasise that a systems medicine approach is particularly attractive to general practitioners – who I believe have always tacitly embraced the notion and practiced accordingly – it must be actively promoted by all involved in providing care to individuals and those having the responsibilities to planning and managing health systems.

I have read this submission. I believe that I have an appropriate level of expertise to confirm that it is of an acceptable scientific standard.

## References

[1] LakoffGJohnsenM: Metaphors we live by.2003;London(The University of Chicago Press) :

[2] MacklemPTSeelyA: Towards a definition of life. *Perspect Biol Med*.2010;53(3) : 10.1353/pbm.0.0167 330-40 10.1353/pbm.0.0167 20639603

[3] JylhäM: What is self-rated health and why does it predict mortality? Towards a unified conceptual model. *Soc Sci Med*.2009;69(3) : 10.1016/j.socscimed.2009.05.013 307-16 10.1016/j.socscimed.2009.05.013 19520474

[4] JadadARO'GradyL: How should health be defined?. *BMJ*.2008;337:a2900 1907366310.1136/bmj.a2900

[5] LewisS: Understanding Health by Building Better Bio-Medical Models, in Handbook of Systems and Complexity in Health (Editors JP Sturmberg and CM Martin). *Springer:New York*.2013;

[6] SturmbergJP: The personal nature of health. *J Eval Clin Pract*.2009;15(4) : 10.1111/j.1365-2753.2009.01225.x 766-9 10.1111/j.1365-2753.2009.01225.x 19674233

[7] SturmbergJP: Health: A Personal Complex-Adaptive State, in Handbook of Systems and Complexity in Health(Editors: JP Sturmberg and CM Martin). *Springer: New York*.2014;231-242

[8] SturmbergJPTopolskiSLewisS: Health: A Systems- and Complexity-Based Definition, in Handbook of Systems and Complexity in Health(Editors: JP Sturmberg and CM Martin). *Springer: New York*.2013;251-253

[9] MoesM: Plato's conception of the relations between moral philosophy and medicine. *Perspect Biol Med*.2001;44(3) :353-67 1148200510.1353/pbm.2001.0055

[10] HusserlE: The Basic Problems of Phenomenology: From the Lectures, Winter Semester, 1910-1911. *Springer: Dordrecht, The Netherlands*.2006;

[11] ParsonsT: The Social System. *Glencoe: Free Press*.1951;

[12] DubosR: The Mirage of Health. *London: Allen and Unwin*.1960;

[13] MaslowHA: Toward a Psychology of Being. *New York: Van Nostrand Reinhold Company*.1968;

[14] TissueT: Another look at self-rated health among the elderly. *J Gerontol*.1972;27(1) :91-4 500919410.1093/geronj/27.1.91

[15] KelmanS: The social nature of the definition problem in health. *Int J Health Serv*.1975;5(4) :625-642 123044110.2190/X5H6-TC5W-D36T-K7KY

[16] IllichI: Limits to Medicine. Medical Nemesis: The Expropriation of Health. *London: Marion Boyars Book*.1976;

[17] AntonovskyA: Health, Stress and Coping. *San Francisco: Jossey-Bass*.1979;

[18] ReidJ: Body, land and spirit. *St Lucia:Queensland University Press*.1984;

[19] SeedhouseD: Health: The Foundations for Achievement. *New York: John Wiley and Sons*.1986;

[20] World Health Organisation: Ottawa Charter for Health Promotion. First International Conference on Health Promotion. Ottawa, 21 November 1986.1986; Reference source

[21] IngstadBFugelliP: “Our Health Was Better in the Time of Queen Elizabeth”: The Importance of Land to the Health Perception of the Botswana San, in Updating the San: Image and Reality of an African People in the 21st Century (Senri Ethnological Studies No. 70), (Editors: RK Hitchcock et al). *Senri; National Museum of Ethnology: Osak*.2006;

[22] HartJT: Expectations of health care: promoted, managed or shared?. *Health Expect*.1998;1(1) :3-13 1128185710.1046/j.1369-6513.1998.00001.xPMC5081066

[23] ColeSW: Human social genomics. *PLoS Genet*.2014;10(8) : 10.1371/journal.pgen.1004601 e1004601 10.1371/journal.pgen.1004601 25166010PMC4148225

[24] GohKIChoiIG: Exploring the human diseasome: the human disease network. *Brief Funct Genomics*.2012;11(6) : 10.1093/bfgp/els032 533-42 10.1093/bfgp/els032 23063808

[25] SturmbergJPBennettJMMartinCMPicardM: “Multimorbidity” as the Manifestation of Network Disturbances. Implications for whole person care (In Press). *Journal of Evaluation in Clinical Practice*.2016;10.1111/jep.1258727421249

[26] FernandezASturmbergJLukersmithSMaddenRTorkfarGColagiuriRSalvador-CarullaL: Evidence-based medicine: is it a bridge too far?. *Health Res Policy Syst*.2015;13: 10.1186/s12961-015-0057-0 66 10.1186/s12961-015-0057-0 26546273PMC4636779

[27] CochraneA: Effectiveness and efficiency. Random reflections on health services. *London: The Nuffield Provincial Hospitals Trust*.1972;

# Peer review: Referee response for version 1

**DOI:** 10.5256/f1000research.8450.r12994

**Published:** 2016-03-31

**Authors:** Amitav Banerjee

**Affiliations:** 1 Department of Community Medicine, Dr. D. Y. Patil Medical College, Pune, India

1. This opinion paper titled, "Understanding the nature of health: New perspective for medicine and public health. Improved wellbeing at lower costs" uses number of illustrative examples on how the Meikirch Model proposed by the lead author in a previous publications can be used as a strategic tool to cope with health and disease burdens in all societies. 
2. The concepts enunciated in the Meikirch Model has some common points in the traditional levels of prevention and epidemiological triad model put forward by Leavell and Clark in the 1940s ^1^. For instance, the biological potential can be compared to the host factors in the epidemiological triad, agent and environment are self explanatory in the epidemiological triad. Environment also is an important component of the Meikirch Model. 
3. While early diagnosis and treatment may be more in line with curative medicine, the level of Tertiary Prevention as proposed by Leavell and Clark has a lot in common with Personally Acquired Potential (PAP) in the Meikirch Model. Psychological Rehabilitation, Social Rehabilitation, Vocational Rehabilitation, Medical Rehabilitation are the activities advocated in Tertiary Prevention which have a lot in common with PAP.
4. Perhaps the authors, may like to bring out these similarities, and in what way the Meikirch Model goes a step further to push the agenda towards empowerment of individuals and communities towards their health promotion by active participation and assuming individual & Community responsibility. This may facilitate a seamless transition in paradigm from the earlier model proposed by Leavell and Clark and the Meikirch Model. 
5. Authors may emphasize how the Meikirch Model has a more positive approach compared to the previous model of health promotion and prevention.
6. In times of rising medical costs the the Meikirch Model can be an important tool and point of reference for policy makers and other stakeholders. 

I have read this submission. I believe that I have an appropriate level of expertise to confirm that it is of an acceptable scientific standard.

## References

[1] LeavellHClarkE: Preventive medicine for the doctor in his community an epidemiologic approach, New York: McGraw-Hill. *1st edition*.1958;
